# The relaxometry hype cycle

**DOI:** 10.3389/fphys.2023.1281147

**Published:** 2023-11-09

**Authors:** Nikola Stikov, Agâh Karakuzu

**Affiliations:** ^1^ Polytechnique Montréal, Montreal, QC, Canada; ^2^ Institut de Cardiologie de Montréal, Université de Montréal, Montréal, QC, Canada; ^3^ Center for Advanced Interdisciplinary Research, Ss. Cyril and Methodius University, Skopje, North Macedonia

**Keywords:** relaxometry, MRI, T1 mapping, T2 mapping, reproducibility, quantitative MRI (qMRI)

## Abstract

Relaxometry is a field with a glorious and controversial history, and no review will ever do it justice. It is full of egos and inventions, patents and lawsuits, high expectations and deep disillusionments. Rather than a paragraph dedicated to each of these, we want to give it an impressionistic overview, painted over with a coat of personal opinions and ruminations about the future of the field. For those unfamiliar with the Gartner hype cycle, here’s a brief recap. The cycle starts with a technology trigger and goes through a phase of unrealistically inflated expectations. Eventually the hype dies down as implementations fail to deliver on their promise, and disillusionment sets in. Technologies that manage to live through the trough reach the slope of enlightenment, when there is a flurry of second and third generation products that make the initial promise feel feasible again. Finally, we reach the slope of productivity, where mainstream adoption takes off, and more incremental progress is made, eventually reaching steady state in terms of the technology’s visibility. The entire interactive timeline can be viewed at https://qmrlab.org/relaxometry/.

## Introduction

### The technology trigger

**FIGURE 1:**
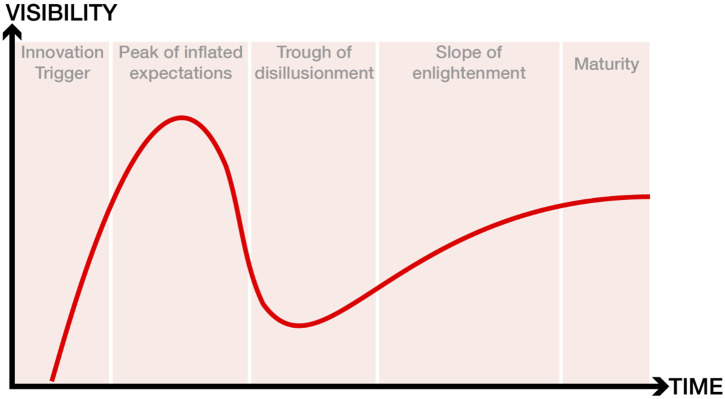
The five stages of the hype cycle. For an interactive version of this figure that features the cited research articles, please visit: https://qmrlab.org/relaxometry/.

In the context of relaxometry, there is no doubt that the technology trigger (Figure 1) is the invention of NMR and its ability to measure relaxation times. Researchers have been using NMR to characterize chemical compounds since the 1930s, but it was the insight of using *in vivo* relaxometry to tell a cancerous tissue sample from a healthy one, that gave birth to MRI. In 1970 Look and Locker published their seminal article on measuring relaxation times with NMR (Look and Locker, 1970), and in 1971 Damadian published a study on the use of NMR-based T1 and T2 values for detecting malignant tumors (Damadian, 1971). Based on this work, he issued a patent application titled “an apparatus and method for detecting cancer in tissue” in 1972, which was accepted in 1974 (Damadian, 1974).

### Inflated expectations

With undeniable insight from the studies of Lauterbur and Mansfield, Damadian’s team built the first human MRI scanner in 1978 and made it commercially available in 2 years. This scanner was essentially a relaxometry device, using T1 and T2 measurements to create a malignancy index and to distinguish between cancerous and non-cancerous tissue. One could argue that the end of the 70s is the peak of inflated expectations, as many researchers believed that the future of MRI is relaxometry. Yet around the same time, GE started manufacturing scanners without paying royalty to Damadian as consideration for his patent. In the decade that followed, GE sold nearly 600 scanners, for which Damadian’s company Fonar filed a patent infringement lawsuit in the late 1990s and was awarded $128,705,766 as compensation for pecuniary damages^1^.

### Trough of disillusionment

It is precisely this lawsuit that resulted in disillusionment about the potential of relaxometry to revolutionize medical imaging. Basically, the court reached the verdict that GE infringed on Damadian’s patent on most counts, except for the count of relaxometry. The original judgment on the verdict reads:

“The Court found that GE had infringed U.S. Patent 3,789,832, MRI’s first patent, which was filed with the U.S. Patent Office in 1972 by Dr. Damadian. The Court concluded that MRI machines rely on the tissue NMR relaxations that were claimed in the patent as a method for detecting cancer, and that MRI machines use these tissue relaxations to control pixel brightness and supply the image contrasts that detect cancer in patients.”

However, the court determined that GE did not infringe on the patent most closely related to relaxometry, as “Fonar failed to establish the existence of standard T1 and T2 values, which are limitations of the asserted claims”. In the years since, MRI manufacturers have insisted that their scanners are not measurement devices and their focus has been on T1-and T2-weighted images that can give visual (qualitative) information about the nature of tissue.

### Slope of enlightenment

The relaxometry community spent the 1980s clawing its way out of the trough of disillusionment. The biggest problem was the time it took to generate a T1 or T2 map, as initial approaches were slow. A relaxometry scan under 10 min was impossible for most body parts, and even those techniques that showed promise were hampered by field inhomogeneities (Levitt and Freeman, 1981), artifacts (Crawley and Henkelman, 1987), and slow post-processing of the data.

The late 80s and early 90s resulted in several relaxometry breakthroughs, such as faster T1 (Fram et al., 1987) and T2 mapping (Poon and Henkelman, 1992), as well as multi-component T2 mapping (Mackay et al., 1994). There were also significant advances in T1rho and T2* mapping. Additionally, several articles were published on standard relaxation times (Gold et al., 2004; Stanisz et al., 2005) that leveled the field while contributing to the slope of enlightenment.

These initial efforts were followed by second-generation relaxometry techniques that provided shorter scan times and larger coverage, such as combining VFA and Look-Locker approaches with SSFP readouts (Messroghli et al., 2004; Deoni et al., 2005; Piechnik et al., 2010; Chow et al., 2014), non-cartesian data acquisitions (Li et al., 2009), and dictionary-based approaches (Marques et al., 2010).

Eventually advances in compressed sensing and dictionary-based approaches resulted in techniques such as MR fingerprinting (Ma et al., 2013) and synthetic MRI (Ji et al., 2022) that allow for simultaneous mapping of T1 and T2, making a full relaxometry scan clinically feasible for most body parts.

These technological innovations were complemented by the efforts of the National Institute of Standards and Technology (NIST) in the US and the National Physical Laboratory (NPL) in the UK to build standardized phantoms for quantitative MRI, as well as the efforts of the Quantitative Imaging Biomarker Alliance (QIBA) to create standards for interpreting quantitative MRI maps. Additionally, the first book on qMRI was published in 2005 (Tofts, 2005).

### Plateau of productivity

2012 saw the publication of a review article on practical medical applications of MR relaxometry (Margaret Cheng et al., 2012). It covered a broad range of relaxometry applications in the brain (multiple sclerosis, stroke, tumors and epilepsy), heart (iron overload, myocardial infarction, edema/inflammation, hemorrhage, methemoglobin, vasodilator function, infarct) and body (iron overload, cartilage disease, injury and infection, cancer). Three case studies with the greatest potential (MS, liver iron and acute myocardial infarction) were covered in-depth. Additionally the article explained the physics behind the pulse sequences and the methodological challenges associated with the technology.

Ten years later we asked the Twitter community to give us an update on what has happened since 2012. We received a number of responses from leaders in the field, and we think it is an informative snapshot of what researchers are most excited about. Most of the themes covered in the 2012 review article are still present, with incremental innovations that confirm that we have reached a plateau of productivity. Below is a Twitter thread^2^ that captures the zeitgeist.

The polled scientists were most excited about the adoption of standardized relaxometry protocols in multiple sclerosis (Sati et al., 2016; Schmierer et al., 2019) and cardiac relaxometry (Teixeira et al., 2016; Messroghli et al., 2017), reproducibility in liver T1 mapping (Tadimalla et al., 2022), T2 and T2* for iron load in Parkinson’s disease (Betts et al., 2016), relaxometry for radiotherapy (Gurney-Champion et al., 2020), and the rise of synthetic MRI (Hagiwara et al., 2017).

The space is short to go through each of these separately, but we have attempted to find the relevant articles and post them online^3^, so feel free to explore and join the conversation. Finally, the last few years saw the update of the Tofts book (Cercignani et al., 2018) and the publication of the most comprehensive textbook on qMRI (Seiberlich, 2020).

## A new trough?

However, we cannot escape the impression that the field has reached another trough. The promise of relaxometry is to generate MR measurements that reproduce across sites. The qMRI community has not delivered on this promise, and clinicians are losing patience. Half a century has passed since the first quantitative T1 (spin-lattice relaxation time) measurements were first reported as a potential biomarker for tumors (Damadian, 1971), followed shortly thereafter by the first *in vivo* quantitative T1 maps (Pykett and Mansfield, 1978) of tumors, but there is still disagreement in reported values for this fundamental parameter across different sites, vendors, and implementations (Stikov et al., 2015; Bojorquez et al., 2017; Hafyane et al., 2018). Add to that the problem of scanner upgrades (Keenan et al., 2019), variability across vendors (Lee et al., 2019), and the inverse crimes that plague AI research in MRI (Shimron et al., 2022). Deep learning in particular cannot resist treating MRIs as measurements, when vendors explicitly tell us not to do that.

## A new slope?

Fortunately, there is hope on the horizon. The qMRI community has ralied behind recent reproducibility efforts (Stikov et al., 2019), pushing for data standards (Karakuzu et al., 2022a), vendor-neutral sequences (Layton et al., 2017; Cordes et al., 2020; Karakuzu et al., 2022b) and open-source processing software (Karakuzu et al., 2020).

2023 is the year when a lot of these efforts are starting to formalize. The International Society for Magnetic Resonance in Medicine (ISMRM) has formed an *ad hoc* committee on standards and, and the MRathon^4^ (a hackathon for MRI professionals) is organizing its second edition focusing on comparable MRI. An idea for a vendor-neutral app store (MRI Fair) won a prize at the 2023 ISMRM shark tank, demonstrating the commercial potential of turning MRI scanners into measurement devices. Finally, we are working on community building by sharing the results of the T1 mapping challenge (Boudreau et al., 2023), publishing the eighth MRM Highlights magazine^5^, and reviving the MRathon Highlights after-party to celebrate reproducibility and transparency in research. There’s never been a more fun time to do relaxometry!

Half a century after its innovation trigger, relaxometry is still full of surprises. There have been peaks and troughs, but the field is slowly maturing thanks to the community contributions to standardization, reproducibility and vendor-neutrality. The relaxometry hype cycle gives us hope that by the end of the decade we will be able to turn the MRI scanner into a true measurement device.

## Data Availability

The original contributions presented in the study are included in the article/supplementary material, further inquiries can be directed to the corresponding author.

## Footnotes

1
https://en.wikipedia.org/wiki/Fonar_Corp._v._General_Electric_Co

2
https://twitter.com/Stikov/status/1521757174967549952

3
https://qmrlab.org/relaxometry/

4
https://mrathon.github.io/

5
https://blog.ismrm.org/2023/06/02/mrm-highlights-magazine-volume-8/
